# Serum biomarkers in Acute Respiratory Distress Syndrome an ailing prognosticator

**DOI:** 10.1186/1465-9921-6-62

**Published:** 2005-06-22

**Authors:** Argyris Tzouvelekis, Ioannis Pneumatikos, Demosthenes Bouros

**Affiliations:** 1 Interstitial Lung Disease Unit, Royal Brompton Hospital, Imperial College, Faculty of Medicine London, UK; 2 Department of Pneumonology, Medical School, Democritus University of Thrace, Greece

**Keywords:** Serum biomarkers, acute respiratory distress syndrome, acute lung injury, cytokines, KL-6, surfactant proteins, adhesion molecules.

## Abstract

The use of biomarkers in medicine lies in their ability to detect disease and support diagnostic and therapeutic decisions. New research and novel understanding of the molecular basis of the disease reveals an abundance of exciting new biomarkers who present a promise for use in the everyday clinical practice. The past fifteen years have seen the emergence of numerous clinical applications of several new molecules as biologic markers in the research field relevant to acute respiratory distress syndrome (translational research). The scope of this review is to summarize the current state of knowledge about serum biomarkers in acute lung injury and acute respiratory distress syndrome and their potential value as prognostic tools and present some of the future perspectives and challenges.

## Introduction

The use of biomarkers in medicine lies in their ability to detect disease and support diagnostic and therapeutic decisions. New research and novel understanding of the molecular basis of the disease reveals an abundance of exciting new biomarkers who present a promise for use in the everyday clinical practice.

The initial evaluation of a serum biomarker concerns its expression in patients with the disease and in normal individuals in order to define sensitivity and specificity. The sensitivity of a test is defined as the proportion of patients with disease having a positive test whereas the specificity is the proportion of patients without the disease who have a negative or normal test. Consequently the serum level of an ideal marker should: 1) increase pathologically in the presence of the disease (high sensitivity), 2) not increase in the absence of the disease (high specificity), 3) relate to the disease burden and extent, 4) change in accordance with the clinical evolution, reflecting the current status of disease, or better 5) anticipate clinical changes, i.e. indicating the presence of relapse before it becomes obvious at a clinical level and finally 6) possess constant serum levels (no major fluctuation) [1].

Additionally, a clinically suitable biomarker should fulfil the following requirements [2]:

1. add independent information about the risk or prognosis

2. account for a large proportion of the risk associated with a given disease or condition

3. be reproducible (as determined by the low coefficient of variation)

4. be sensitive, specific and should present with a high predictive value

5. be of easy and cheap determination

Very few markers present a threshold at which the risk suddenly rises. The interplay between sensitivity and specificity and the nature of the disease under prediction assigns suitable cut-off points. Sensitivity and specificity calculated at various cut-off points give rise to a receiver-operating-characteristic (ROC) curve [2]. A clinically useful biomarker will be one with the largest area under the ROC curve. A number of novel blood biomarkers of lung disease including cytokines, enzymes, adhesion molecules, collagen relevant products and products of type II epithelial cells, have been studied for their clinical applicability.

The scope of this review is based on the fact that although there are numerous published papers investigating the utility of biomarkers in the clinical research field the number of review articles summarizing the current state of knowledge about the clinical applications of these molecules as diagnostic and prognostic tools in the research field relevant to acute respiratory distress syndrome (ARDS) and acute lung injury (ALI) still remains inadequately small.

## Serum biomarkers in Acute Respiratory Distress syndrome

ARDS is a clinical and pathophysiologic entity characterized by severe acute injury, directly or indirectly via the blood, to the endothelial and epithelial surfaces of the lung leading to respiratory failure. The main characteristics of the syndrome are diffuse inflammation and increased microvascular permeability that cause diffused interstitial and alveolar oedema and persistent refractory hypoxemia [3]. Although a variety of insults may lead to ARDS, a common pathway may probably result in the lung damage [4,5]. A complex series of inflammatory events have been recognized during the development of ARDS but the exact sequence of the events remains elusive. Immunological studies investigating bronchoalveolar lavage fluid (BALF) have shed further light into the pathogenetic mechanisms of ARDS [6] and formed the basis of concepts of its immunopathogenesis. A large variety of inflammatory mediators (Table 1) have been found to be elevated in the early phase of ARDS, including lung-specific proteins, endotoxin binding proteins, tumor necrosis-alpha (TNFa), interleukins – (ILs) – 1, 2, 6, 8, 15, chemokines, ferritin, markers of endothelium activation (adhesive molecules and von-Willebrand factor antigen-VWF) as well as markers of neutrophil activation such as matrix metalloproteinases (MMPs) and their inhibitors and leukotrienes [5-7]. The majority of these molecules have features to recommend them as biologic markers in ARDS. Biomarkers have attracted a lot of attention in both ALI and ARDS since they can shed further light into the pathogenesis and pathophysiology of lung injury. Additionally, from a practical point of view, a clinical useful biomarker for ARDS must add information regarding the development of syndrome in at-risk patients that is not apparent from routine examination and investigation. The latter, could help the intensivist to monitor the disease and evaluate or modulate treatments before they have failed. Driven by this perspective idea, many studies have estimated their usefulness as early predictors of ARDS and accurate markers of lung injury before clinical changes can be detected.

**Table 1:** List of studied serum biomarkers in ARDS

**Lung epithelium-specific proteins**	**Surfactant-associated proteins**
	• SP-A
	• SP-B
	• SP-D
	
	**Mucin-associated antigens**
	• KL-6/MUC1
	
**Cytokines**	• IL-1
	• IL-2
	• IL-6
	• IL-8
	• IL-10
	• IL-15
	• TNFa
	
**Other serological parameters**	**Markers of endothelium activation**
	• Adhesion molecules (E, L-selectin, I-CAM-1, V-CAM-1)
	• VWF
	
	**Markers of neutrophil activation**
	• MMP-9
	• LTB4
	**Ferritin**

Abbreviations: ARDS: Acute Respiratory Distress Syndrome, CC16: Clara-cell protein 16, IL: Interleukin, KL-6: Krebs von den Lungen-6, LTB4: Leukotriene B4 MMP-9: Metalloproteinase-9 MUC: Mucin, I-CAM-1: Intercellular Adhesion Molecule-1, sIL-2R: soluble interleukin-2 receptor, V-CAM-1: Vascular Adhesion Molecule-1, VWF: von Willebrand factor antigen

## Cytokines (Tables 2 and 3)

**Table 2:** Studies measuring cytokines in patients with or at risk for ARDS

**Investigator**	**Patients Controls**	**Biomarker / Summary**	**ROC curve analysis Cut-off values**	**Specificity – Sensitivity PPV – NPV**	**Limitations**
Pinsky et al. ^13^	52 at risk	Relation of IL-6 and TNF plasma levels to multiple-system organ failure and mortality	No	Not estimated	Small number of patients No ROC curve analysis / cut-off levels No serial measurement
Takala et al. ^14^	20 at risk 56 controls	IL-6 and IL-8 plasma levels predict organ failure in community-acquired septic shock	No	Not estimated	Small number of patients No ROC curve analysis / cut-off levels Poor discriminative value of serum biomarkers per se
Calandra et al. ^15^	70 at risk	IL-6 plasma levels do not predict the outcome in at risk patients for ARDS	No	Not reported	Small number of patients No ROC curve analysis
Kiehl et al. ^16^	19 at risk	TNFa, IL-6, IL-8 plasma levels fail to associate with severity and course of ARDS in leukocytopenic patients	No	Not estimated	Small number of patients No ROC curve analysis / cut-off levels Leukocytopenic patients
Meduri et al. ^18^	27 ARDS	TNFa, IL-1β, IL-2, IL-4, IL-6, IL-8 Superiority of IL-1β and IL-6 plasma levels in monitoring disease activity over commonly applied clinicophysiologic parameters.	Yes TNFa: 400 pg/ml IL-1β: 400 pg/ml IL-2: 200 pg/ml IL-4: 200 pg/ml IL-6: 400 pg/ml IL-8: 400 pg/ml	TNFa: 89-50-85-57% IL-1β: 78-83-83-78% IL-2: 89-83-90-80% IL-4: 89-50-85-57% IL-6: 77-75-81-70% IL-8: 66-50-66-50%	Small number of patients Perspective study Overlap of cytokine levels between survivors and non-survivors Heterogeneity of studied population Definition criteria of ARDS
Agouridakis et al. ^19^	8 ARDS^a^ 26 at risk	Association between increased levels of IL-2 and IL-15 and outcome in patients with early ARDS	Yes IL-2: 173 pg/ml IL-15: 250 pg/ml	IL-2: 100-100-100-100% IL-15: 100-100-100-100%	Small number of patients Limited number of studied molecules

Abbreviations: ARDS: Acute Respiratory Distress Syndrome, BALF: Bronchoalveolar lavage Fluid, IL: Interleukin, NPV: Negative Predictive Value, PPV: Positive Predictive Value, ROC: Receiver Operating Characteristic, sIL-2R: soluble interleukin-2 receptor, TNFa: Tumor Necrosis Factor-alpha

^a: ^Use the American European Consensus Conference definitions

**Table 3:** Studies measuring cytokines in patients with or at risk for ARDS

**Investigator**	**Patients Controls**	**Biomarker / Summary**	**ROC curve analysis Cut-off values**	**Specificity – Sensitivity PPV – NPV**	**Limitations**
Lesur et al. ^20^	19 ARDS^a^ 14 at risk 20 controls	Association of early low serum IL-2 levels with the patients' survival	No	Not estimated	Small number of patients No ROC curve analysis / cut-off levels No serial measurement Discrepancies in serum and BALF IL-2 levels Limited number of studied molecules
Parsons et al. ^23^	77 at risk	Association of serum IL-1ra, IL-10 levels with the disease outcome	No	Not estimated	No ROC curve analysis / cut-off levels Poor predictive value for ARDS development Limited number of studied molecules
Takala et al. ^24^	52 at risk 9 ARDS^a^ 45 controls	IL-8, IL-6, sIL-2R, E-selectin, procalcitonin Persistent elevation of inflammatory markers in patients with ALI precedes its clinical diagnosis	No	Not estimated	Small number of non-survivors No ROC curve analysis / cut-off levels Poor predictive value for ARDS development
Bouros et al. ^25^	32 ARDS^a^ 27 at risk	IL-4, IL-6, IL-6r, IL-8, IL-10 High prognostic value of all the inflammatory markers in assessing the outcome in patients with or at risk for ARDS	Yes IL-4: 84 pg/ml IL-6: 160 pg/ml IL-6r: 18 pg/ml IL-8: 2340 pg/ml IL-10: 98 pg/ml	IL-4: 78-100-81-100% IL-6: 59-96-69-94% IL-6r: 78-76-76-78% IL-8: 93-96-92-96% IL-10: 96-92-96-93%	Small sample size No serial measurement Grouping and definition criteria
Schutte et al. ^26^	30 ARDS^a^ 44 at risk 17 controls	IL-6, IL-8, TNFa Serum levels of IL-6 and IL-8 in ARDS and/or severe pneumonia, differentiate these entities from cardiogenic pulmonary oedema	No	Not estimated	Small number of patients Weak correlations with clinical variables No definitive predictive value for outcome Overlap of cytokine levels between survivors and non-survivors No ROC curve analysis / cut-off levels
Bauer et al. ^27^	46 ARDS^a^ 20 at risk 10 controls	IL-6, IL-1b, TNFa serum levels associate better with the degree of lung injury rather than clarify its specific aetiology	No	Not estimated	Corticosteroid treatment Inconclusive prognostic value No serial measurement Limited number of studied molecules

Abbreviations: ARDS: Acute Respiratory Distress Syndrome, BALF: Bronchoalveolar lavage Fluid, IL-1ra: Interleukin-receptor antagonist, NPV: Negative Predictive Value, PPV: Positive Predictive Value, ROC: Receiver Operating Characteristic, sIL-2R: soluble interleukin-2 receptor, TNFa: Tumor Necrosis Factor-alpha

^a: ^Use the American European Consensus Conference definitions

Cytokines are widely recognized as mediators of an inflammatory response. Their discovery has stimulated multidisciplinary investigation to elucidate the role of these mediators in the injury and repair processes of human disease. In the lung, they are produced either by local resident cells such as alveolar macrophages, pneumocytes, endothelial cells and fibroblasts or by cells such as neutrophils, lymphocytes and platelets arriving to the lung in response to local or systemic injury [8,9]. Cytokines are involved both in the early (TNFa, IL-1, 2, 6, 8, 15) and late phase (IL-4) of inflammation and have been shown unequivocally to be of crucial importance in the pathophysiology of septic shock, a condition frequently culminating to ARDS [10,11].

Studies have demonstrated that in ARDS patients detectable cytokine serum levels are closely related to the disease severity and mortality [11,12], suggesting a potential role in reflecting the severity of the lung injury. Moreover, humoral (IL-6, IL-8) and cellular markers (CD 11b) of systemic inflammation have been delineated to identify patients with septic shock at risk for organ failure, culminating to a fatal outcome [13,14]. However, their monitoring and prognostic value in septic-shock patients still remains controversial. Calandra et al. [15] stated that serum cytokines could not be used as a routine laboratory test to predict the outcome in septic-shock patients. In addition, Kiehl et al. [16] failed to prove usefulness of plasma cytokines measurement for the evaluation of severity and course of ARDS in a small cohort of leukocytopenic septic-shock patients. In the same study, estimation of BALF levels appeared to differentiate between responders and non-responders to treatment before clinical differences become apparent. Nonetheless, it should be noted that the small sample size, the contradictive results, the lack of standardization techniques and uniform definitions for ARDS and at risk patients, together with the heterogeneity of the syndrome and the patients studied generate major concerns regarding the reproducibility and the reliability of the data presented. Consequently, there is a need for further large scale investigations in the context of appropriate clinical trials for any meaningful conclusions to be reached.

Evidence from preceding studies [11-13,16] give credence to the view that the inability of the lung to repair after ALI is the result of a persistent inflammatory stimulus that ultimately leads to an unfavorable outcome [17]. Fueled by this prospect, Meduri and coworkers [18] indicated a consistent, efficient and independent predictive value for IL-1β and IL-6 serological concentrations over time in a small cohort of patients with severe ARDS. They generated ROC curve analysis and demonstrated a clear superiority of inflammatory cytokines in monitoring disease activity over commonly applied clinicophysiologic parameters. However, this study exhibited substantial weaknesses including the retrospective analysis, the small sample size and the overlapping results between survivors and non-survivors. These observations coupled with the heterogeneity of the disease and the evidence that elevated serum cytokines may reflect the increased production or decreased clearance and not the disease activity pose major limitations to the aforementioned findings.

In another study, Agouridakis et al. [19] evaluated both the prognostic and predictive significance of IL-2 and IL-15 for the development and outcome of patients at risk who developed ARDS or patients at risk who never developed ARDS, respectively. They applied ROC curve analysis and showed an excellent predictive value of cytokine plasma levels in terms of specificity and sensitivity compared to those observed in BALF. The most remarkable ascertainment of this study was the emergence of the discriminative usefulness of elevated IL-2 and IL-15 serological concentrations in patients with ARDS or at risk for ARDS. Nevertheless, the small number of patients enrolled combined with the lack of serial measurements throughout the clinical course of the disease and the causal diversity of the syndrome render major uncertainty to these findings.

On the contrary with other analyses [16,18], Lesur et al [20] found lower blood IL-2 levels in patients with ARDS compared to those that never developed the syndrome. In addition, evidence of this study regarding the strong association of early low serum IL-2 levels with the patients' survival corroborated earlier findings [16]. Potential criticism of this study include the small number of patients recruited, the absence of multiple time-point evaluation of the cytokine plasma concentrations and more importantly opposite and disproportional fluctuations of IL-2 content in serum and BALF in patients with or without ARDS.

The role of several inflammatory cytokines in monitoring the disease activity and predicting the survival in patients with ARDS has aroused increasing attention the past decade. One of the most intriguing aspects of the application of these biomarkers in the daily clinical practice is the early detection of patients admitted to the intensive care unit (ICU) that will develop ARDS. This approach will allow anti-inflammatory and other supportive treatments to be evaluated or eventually modified before they have failed. Predictive levels of inflammatory cytokines (IL-1, IL-2, IL-6, IL-8) for ARDS development in at risk patients have been extensively reported with controversial results [16,17,21,22]. The importance of considering inflammatory constituents of serum in patients at risk for ARDS was initially raised by Parsons et al [23]. Authors conducted a large prospective analysis and demonstrated that although immunological parameters (IL-1ra and IL-10) were elevated in patients at risk for ARDS and exhibited a remarkable association with the disease outcome, none of these could predict the development of the syndrome.

These observations were extended by the results of Takala et al. [24] who showed that serum levels of inflammatory mediators albeit their persistent elevation in patients with unresolving ALI, preceding its clinical diagnosis, were of poor discriminative value in patients with ALI that did or did not develop ARDS. Nevertheless, these findings provided us with useful knowledge about the inflammation marker profile on the days preceding diagnosis of ARDS, indicating a potential relation of sustained inflammatory response with a poor outcome.

With this aim in mind, Bouros et al. [25] measured prospectively a slew of cytokines in the serum and BALF in ICU patients to identify predictive factors for the course and outcome of ARDS. The most remarkable result of this analysis was that almost all serum molecules studied showed a high prognostic value in assessing the outcome in patients with or at risk for ARDS. However, laboratory parameters failed to prove a positive correlation with the prediction of ARDS development evidence consistent with earlier studies [23,24]. Moreover, major caveats that should be taken under consideration include the limited number of patients, the lack of sufficient follow-up serum data and the marked causal heterogeneity of the syndrome that could be a reason for the contradictive results reported in previous studies [20,23-25]. Further prospective studies with sufficient statistical power are required to validate these results and ameliorate the predictive role of circulating inflammatory mediators in patients at risk for ARDS.

Although the role of cytokines in the pathogenesis of ARDS has been extensively investigated, their importance in the differential diagnosis has not been clearly defined. It is widely accepted that numerous insults may lead to ARDS following a common pathway. Furthermore, a variety of conditions including severe pneumonia imitate clinical and radiological manifestations of ARDS and thereby it is often difficult to differentiate them. However, this would be a fruitful application because the treatment of these conditions differs considerably. Many groups of investigators have attempted to produce a discriminative systematic inflammatory profile and although much good work has been done towards this direction, the results still remain controversial.

Schutte and co-workers [26] provided us with a really well done and heavily informative paper concerning the systemic cytokine profile in patients with ARDS, severe pneumonia and cardiogenic pulmonary oedema. Authors found remarkably and consistently elevated serum levels of IL-6 and IL-8 in ARDS and/or severe pneumonia, differentiating these entities from cardiogenic pulmonary oedema. Nevertheless, they were unable to separate the various entities of ARDS and states of severe pneumonia based solely on alterations in the immunomodulatory pattern.

To streamline these observations, Bauer et al. [27] tested the potential of inflammatory markers (TNFa, IL-1β, IL-6) to differentiate between these two diseases. Results in harmony with the previous study [26] demonstrated higher TNFa serological concentrations in patients with ARDS from the remaining populations. However, they revealed the ability of immunological parameters to associate better with the degree of lung injury rather than clarify its specific aetiology. No clear relationship between serological data and patients' survival was observed. In addition, this data exhibits major limitations, including the absence of uniform methodology (use of corticosteroid treatment in some of the patients), serial measurements and the lack of knowledge regarding serum alterations in other components of the inflammatory network (Tables 2 and 3).

## Other serological parameters

### Markers of endothelium activation (Tables 4 and 5)

**Table 4:** Studies measuring markers of endothelium activation in patients with or at risk for ARDS

**Investigator**	**Patients Controls**	**Biomarker / Summary**	**ROC curve analysis Cut-off values**	**Specificity – Sensitivity PPV-NPV**	**Limitations**
Donnelly et al. ^39^	82 at risk 14 ARDS^a^ 62 controls	E-selectin levels were not correlated with ARDS development and patients' mortality. L-selectin levels exhibited a significant prognostic value	No	Not estimated	Heterogeneity of studied population (trauma-sepsis) No ROC curve analysis / cut-off levels No serial measurement
Boldt et al. ^40^	50 at risk	Constantly lower E-selectin, ICAM-1 and VCAM-1 levels in survivors experiencing polytrauma than in nonsurvivors	No	Not estimated	Small sample size Causal diversity of patient group No definitive association with patients' mortality No ROC curve analysis / cut-off levels
Cowley et al. ^41^	40 SIRS 85 controls	Superiority of E-selectin plasma levels in predicting organ dysfunction and death patients with SIRS comparing to ICAM-1	No	Not estimated	Small number of patients Causal diversity of patients studied No ROC curve analysis / cut-off levels
Sessler et al. ^43^	25 at risk 12 controls	Association of elevated ICAM-1 Sequential plasma levels with the severity of shock	Yes ICAM-1: 715 ng/ml (predicting survival)	Not reported	Small sample size Heterogeneity of studied population Inconclusive association with disease Severity
Kayal et al. ^44^	32 at risk 9 controls	Cut off values of E-selectin, ICAM-1 and VWF serum levels predicted survival outcome	Yes E-selectin: 128 ng/ml ICAM-1: 715 ng/ml VWF: 717%	E-selectin: 73-80-67-85% ICAM-1: 80-90-75-92% VWF: 87-8080-87%	Small number of patients Most of the patients developed secondary ALI
Agouridakis et al. ^45^	23 ARDS^a^ 42 at risk	TNFa, IL-1, ICAM-1, VCAM-1 ICAM-1 and VCAM-1 showed a high NPV for ARDS development Correlation with the disease outcome None of the studied markers was an independent factor for ARDS development	Yes TNFa: 325 pg/ml IL-1: 225 pg/ml ICAM-1: 300 pg/ml VCAM-1: 260 pg/ml	For ARDS development TNFa: 62-75-38-89% IL-1: 58-88-39-94% ICAM-1: 69-75-42-90% VCAM-1: 73-88-50-95%	Small sample size No serial measurement None of the studied markers was an independent factor for ARDS development

Abbreviations: ALI: Acute Lung Injury, ARDS: Acute Respiratory Distress Syndrome, ICAM-1: Intercellular Cell Adhesion Molecule-1, IL: Interleukin, ROC: Receiver Operating Characteristic, SIRS: Systematic Inflammatory Response Syndrome, TNFa: Tumor Necrosis Factor-alpha, VCAM-1: Vascular Cell Adhesion Molecule-1, VWF: von Willebrand factor antigen, ^a: ^Use the American European Consensus Conference definitions

**Table 5:** Studies measuring markers of endothelium activation in patients with or at risk for ARDS

**Investigator**	**Patients Controls**	**Biomarker / Summary**	**ROC curve analysis Cut-off values**	**Specificity – Sensitivity PPV-NPV**	**Limitations**
Rubin et al. ^47^	45 at risk	Elevated plasma VWF is an early predictor of ALI in nonpulmonary sepsis syndrome	Yes VWF: 450%	77-87-80%	Small sample size 25% of patients had already lung injury at the time sepsis was diagnosed Exclusion of patients who developed ALI from a primary pulmonary source VWF levels measured by an old assay
Ware et al. ^48^	51 ALI/ ARDS^a^ 4 controls	VWF is an independent predictor of hospital mortality in patients with ALI	No VWF:450%	91-44-83-62%	Inadequate sample volume Heterogeneity of studied population No ROC curve analysis
Ware et al. ^49^	559 ALI/ ARDS^a^	Significant correlation of elevated VWF plasma levels with mortality, duration of unassisted ventilation and organ failures. No differences of VWF levels between septic and non septic patients	No	Not estimated	Not definitive association with patients' mortality Lack of knowledge regarding the cellular source and the mechanisms of elevated VWF serum levels No ROC curve analysis / cut-off values
Moalli et al. ^50^	35 at risk 10 ARDS 9 controls	VWF levels were higher in ARDS compared with at risk VWF levels are not helpful in predicting ARDS development	No	Not reported	Limited number of patients No ROC curve analysis / cut-off values
Moss et al. ^51^	96 at risk	VWF is not predictive of development of ARDS	Yes VWF:273% VWF:399%	47-70% 52-64%	Causal diversity of patients studied No definitive relation with disease severity
Sabharwal et al. ^52^	22 ARDS 21 at risk	No significant association of VWF blood levels with patients' mortality	No	Not estimated	Small sample size Retrospective study No ROC curve analysis / cut-off values
Bajaj et al. ^53^	18 ARDS 15 at risk 27 controls	Serum VWF levels were non-useful markers for predicting ARDS in at risk patients	Yes VWF: 300%	71-62-34%	Limited number of patients No serial measurement Coexisting multisystem organ failure Heterogeneity of studied population
Moss et al. ^54^	55 at risk 14 ARDS 11 controls	ICAM-1, E-selectin, VWF Degree of endothelial activation varied in patients at risk for ARDS from different etiologic factors	No	Not estimated	Small sample size Heterogeneity of studied population

Abbreviations: ALI: Acute Lung Injury, ARDS: Acute Respiratory Distress Syndrome, ICAM-1: Intercellular Cell Adhesion Molecule-1, NPV: Negative Predictive Value, PPV: Positive Predictive Value, ROC: Receiver Operating Characteristic, VCAM-1: Vascular Cell Adhesion Molecule-1, VWF: von Willebrand factor antigen

^a: ^Use the American European Consensus Conference definitions

The pathophysiologic sequence characterizing ALI involves apart from cytokine, free radical, proteases and aracidonic acid metabolites release, the endothelial and neutrophil activation which initiate a cascade of leukocyte-endothelium interactions and adhesions. This is followed by transendothelial migration of neutrophils and release of their cytotoxic products, ultimately resulting to microvascular and tissue injury [28].

Adhesion of neutrophils to the endothelium is regulated by at least three adhesion molecule families including selectins (E, L and P), integrins and the immunoglobulin superfamily (intercellular adhesion molecule- ICAM-1 and vascular cell adhesion molecule-VCAM-1) and by chemotactic signals [29,30]. Initial interactions of leukocytes and the endothelium are mediated by members of the selectin family inducing (loose) contact with the endothelium also known as *rolling*, followed by firm adhesion requiring members of the integrin (β2) and immunoglobulin family (ICAM-1) [31,32].

In recent years, soluble isoforms of some of these molecules {soluble-(s)-E-selectin, sICAM-1, sVCAM-1} have been detected in the circulating blood under various inflammatory conditions [33-35]. Mechanisms that could potentially explain an increase in circulating adhesion molecules include cytokine-induced (IL-1, TNFa) overexpression by the endothelial cells, increased proteolytic cleavage of endothelial-bound adhesion molecules secondary to endothelial damage or both [33,35]. One attractive feature of these molecules and mostly E-selectin is that since their expression is almost restricted to stimulated endothelial cells [32] their presence in serum should potentially reflect the state of endothelium in disease and subsequently the disease severity in ALI.

Other potential markers of endothelial cell injury that were delineated to shed further light into the pathophysiologic process of ALI include von-Willebrand factor antigen (VWF), a macromolecular antigen that is produced predominantly by endothelial cells and to a lesser extent by platelets and megakaryocytes [36]. Endothelial perturbation (as in at risk state) or injury (as in ARDS) results to the release of VWF from preformed stores into the circulation [37,38]. Therefore, it appears that circulating VWF concentrations may serve as a suitable predictive marker for development of ARDS in patients at risk.

So far, the potential usefulness of adhesion molecules and other markers of endothelial cell damage in reflecting the severity of endothelial damage and predicting the development or the final outcome of the disease is a subject of ongoing controversy. One of the first and most informative studies addressing this important issue was conducted by Donnelly et al [39]. Authors demonstrated in a large cohort of patients at risk for ARDS, that mean circulating levels of sE-selectin were not correlated with subsequent ARDS development and patients' mortality. However, low values of sL-selectin exhibited a significant prognostic value. In contrast, Boldt et al. [40] studying the behaviour over 5 d of adhesion molecules (sE-selectin, sICAM-1 and sVCAM-1) in subjects experiencing polytrauma found constantly higher levels in nonsurvivors. In accordance to these findings, Cowley and colleagues [41] showed a superiority of sE-selectin plasma levels in predicting organ dysfunction and death in a group of patients with systemic inflammatory response (SIRS) comparing to sICAM-1 peripheral concentrations. This indicated that measurement of adhesion molecules could serve to advantage in the management of patients with sepsis. Nonetheless, the heterogeneity of patients studied (septic shock and polytraumatic) may justify these controversial results, since E-selectin expression has been found much greater in septic than in traumatic shock in experimental models [42].

Moreover, the relationship between the consequences of sepsis (organ failure, mortality) and blood levels of potential markers of endothelial-cell activation was strengthened by Sessler and co-workers [43]. Results from this study focusing on sICAM-1 sequential plasma levels, were suggestive of a strong association between the severity of shock (as determined by the presence of hypotension and the requirement of vasoactive drugs) and the circulating concentrations of the marker. The aforementioned observations were further confirmed by the study of Kayal et al [44]. Cut off values for three markers of endothelial activation were determined prospectively by ROC curve analysis and clearly predicted survival outcome with high sensitivity and specificity in a limited number of at-risk patients with secondary ALI.

Despite that the role of soluble adhesion molecules in other inflammatory conditions strongly associated with ARDS [40-44] is well known, their value as markers of the disease progression and mortality has not been extensively studied in ARDS patients. To streamline these observations, Agouridakis et al. [45] scrutinized the role of two adhesion molecules (ICAM-1, VCAM-1) in parallel with proinflammatory cytokines in predicting the ARDS development and relating to the disease outcome. None of the studied mediators was found to be an independent factor for ARDS development, whereas both groups of molecules exhibited a considerable negative predictive value for ARDS development both in serum and BALF. Additionally, ROC curve analysis showed a clear superiority of plasma parameters in correlating with the disease outcome compared with BALF molecules. Further studies with serial BALF and serum measurements should be designed to elucidate the exact role of these markers over time in reflecting the disease behaviour and predicting the likelihood of progression.

Elevated circulating concentrations of VWF in patients with ALI/ARDS were first reported in 1982 [46]. Their potential significance in predicting ARDS development was first demonstrated by Rubin and colleagues [47] who found that increased plasma levels of this marker exhibited a high predictive value both for the development of ARDS and for identifying patients with nonpulmonary sepsis who were unlikely to survive. However, authors did not use the uniform criteria for the definition of ARDS and risk state [3], evidence that poses major limitations to the results of the study.

Furthermore, in an aforementioned study, Kayal et al. [44] in parallel with other findings reported a marked and independent association of circulating VWF with the disease severity as assessed by other commonly applied clinical variables. These findings were further supported by a single-center study from Ware et al [48]. They conducted the first comparative study of VWF concentrations in both plasma and edema fluids of patients with early ALI from a variety of causes and reported that serum VWF levels were an independent predictor of hospital mortality and were associated with longer duration of mechanical ventilation. Potential criticisms of this study was the implementation of high tidal volume ventilation that possibly increased systemic endothelial activation, the small sample size, the lack of sequential measurement and the fact that the studied biomarker appeared not to be endothelial-specific since it is produced in small amounts by platelets [36].

To streamline these observations and ameliorate potential hardships the same group of authors {Ware et al. [49]} carried out a multicenter study of 559 patients with ALI and ARDS which was recently published. In accordance with earlier studies [48], a significant correlation of elevated VWF plasma levels with adverse outcomes, including mortality, duration of unassisted ventilation and organ failures was pointed out. Intriguingly, authors demonstrated for the first time a negative association between markers of endothelial activation and presence or absence of sepsis, supporting the hypothesis that ALI might be an independent cause of systemic endothelial activation and injury. Finally, in the same study no modulation of plasma VWF concentrations by protective mechanical ventilation was observed. Despite the remarkable power of the presented findings, there are substantial weaknesses that deserve further investigations including the inconclusive analysis of the plasma VWF levels associated with patients' mortality, the lack of definite knowledge regarding the source of VWF production, and the mechanisms leading to increased peripheral concentrations since the latter also could reflect decreased clearance from the circulation.

Subsequent data derived from other studies [50-54] was rather contradictive and controversial. Even though, Moalli et al. [50] found a poor predictive value of serum VWF levels for the development of ARDS in a group of at risk patients, the biomarker concentrations were correlated with the disease severity,. Similarly, Moss et al. [51] plotted ROC curves and concluded that in patients at risk for ALI/ARDS from multiple causes, serum VWF levels failed to reliably discriminate which patients would develop ARDS. The evidence was further validated by Sabharwal et al [52]. Authors conducted the first study comparing plasma levels between survivors and nonsurvivors in a group of patients both at risk for and with established ARDS and observed no significant association of VWF blood levels with patients' mortality ;predictive value of the marker was not reported. In agreement with the previous study, a study from the same group of scientists {Bajaj et al. [53]} using standard criteria for the definition of ARDS and at risk state [3] demonstrated the inability of three endothelial-specific proteins including VWF to predict the progression of ARDS in at risk patients. Nonetheless, major caveats that should be addressed include the fact that many at-risk patients had already some degree of ALI, the lack of serial measurement that could potentially show a trend towards prediction of ARDS development and the causal diversity of patients examined that could possibly affect the results of the study. The latter limitation was addressed by Moss et al. [54] who established that the degree of endothelial activation as determined by the plasma levels of VWF (higher in subjects with sepsis than patients with trauma) is not uniform in all patients at risk for developing ARDS.

Accumulated evidence from the preceding studies suggest that the etiologic diversity of patients enrolled renders major uncertainty to the reliability of the results and highlights the necessity for further prospective studies using standard criteria for the definition of ARDS and analyzing well defined and uniform group of at risk patients in order to produce knowledge of high scientific rigidity. Direct comparison of the different studies is difficult and in a way meaningless because of the use of varying definitions for ARDS and at-risk patients as well as the inclusion of different patient populations, in which of them some degree of ALI was probably already present (Tables 3a and 3b).

### Markers of neutrophil activation (Table 6)

**Table 6:** Studies measuring markers of neutrophil activation and ferritin in patients with or at risk for ARDS

**Investigator**	**Patients Controls**	**Biomarker / Summary**	**ROC curve analysis Cut-off values**	**Specificity – Sensitivity PPV – NPV**	**Limitations**
Pugin et al. ^60^	31 at risk 23 ARDS^a^	IL-8, MMP-2, MMP-9 Plasma levels of inflammatory activity are not useful markers in differentiating permeability from hydrostatic pulmonary edema	No	Not estimated	Measurement of circulating proinflammatory cytokines without the appreciation of their inhibitors or receptor antagonists is misleading mainly due to a possible neutralization Small number of patients No ROC curve analysis / cut-off values
Amat et al. ^64^	21 ARDS^a^ 14 at risk	Strong association of LTB4 and IL-8 serum levels with the patients' survival.	Yes LTB4: 14 pmol/ml IL-8: 150 pmol/ml	LTB4 + IL-8: 88-70-85-75% (markers of mortality rate) LTB4 : 85-72-20-98% (marker of ARDS development)	Small sample size Lack of adjustment with the disease severity Inability of LTB4 plasma levels to be an independent predictive marker
Connelly et al. ^66^	75 at risk 8 ARDS^a^	Serum ferritin is a sensitive and specific predictor of ARDS development	Yes Ferritin (male):270 ng/ml Ferritin (female): 680 ng/ml	71-83-86-67% 90-60-82-75%	Limited number of ARDS patients Heterogeneity of studied population
Sharkey et al. ^67^	42 at risk 16 ARDS^a^	Correlation of ferritin plasma levels with the development of ARDS, multiple organ failure and severity of lung injury	Yes Ferritin (male):270 ng/ml Ferritin (female): 680 ng/ml	64-73-75-62% 92-60-95-75%	Inadequate sample volume Not specific cut-off values Elevated serum levels may reflect a systemic response to a risk factor

Abbreviations: ARDS: Acute Respiratory Distress Syndrome, IL: Interleukin, LTB4: Leukotriene B4, MMP-Metalloproteinase, NPV: Negative Predictive Value, PPV: Positive Predictive Value, ROC: Receiver Operating Characteristic, ^a: ^Use the American European Consensus Conference definitions

Generally, it is strongly believed that ARDS arises as a result of tissue injury secondary to sequestration of inflammatory cells, tissue invasion, and secretion of cytotoxic products. Neutrophils have received much attention as key part of this process. Although ARDS has been described in neutropenic patients [16,55] there is increased evidence implicating neutrophils in most cases of ARDS. They have been reported by several studies [56,57] to exert an important role in the early phase of ALI characterized by architecture remodeling, surfactant and epithelial toxicity. They use a wide array of enzymes during the process of transmigration through biological membranes such as alveolar-capillary barrier [58]. These enzymes include, metalloproteinases (MMPs) such as MMP-9 also called gelatinase B which is secreted from preformed neutrophil granules in response to a variety of stimuli including proinflammatory cytokines (IL-8, TNFa). MMP-9 is secreted as a zymogen, and then activated by a variety of other proteases such as elastase, and plays a crucial role in digesting basement membranes [58,59]. Therefore, it has been speculated that metalloproteinases probing aspects of the inflammatory response could be utilized as markers of neutrophil activation and subsequently to reflect disease activity and severity, shedding further light into the pathogenesis of ARDS.

Fueled by this prospect, Pugin et al. [60] compared the concentrations of proinflammatory cytokines and collagenases in serum and pulmonary oedema fluids in a small group of patients with ARDS and hydrostatic oedema from congestive heart failure. Authors concluded that elevated pulmonary oedema levels of these mediators could differentiate between these conditions, whereas plasma levels of proinflammatory and metalloproteinase activity proved to be of poor discriminative value. The latter observation mirrors the hypothesis that the inflammatory response characterizing ARDS patients is well compartmentalized, with little spillover into the circulation and that the measurement of circulating proinflammatory cytokines without the appreciation of their inhibitors or receptor antagonists is misleading mainly due to a possible neutralization.

To gain a more comprehensive understanding on the role that neutrophils exhibit during the inflammatory cascade resulting to ALI, investigators scrutinized the utility of other chemotactic agents including leukotrienes (LTs). LTs (B4, C4, D4, E4) exert a synergistic role with IL-8 in the neutrophil influx and activation leading to a massive recruitment of neutrophils and to a catastrophic inflammatory response. Their BALF levels have been found elevated in patients with ARDS and their involvement in the alterations of microvascular permeability correlated with the accumulation of pulmonary oedema has been suggested [61-63].

Moreover, Amat et al [64] utilizing ROC curve analysis demonstrated that LTB4 plasma levels could serve as a valuable predictive marker of ARDS in terms of specificity and sensitivity. In the same study, authors performed serial measurement and reported a strong association of both LTB4 and IL-8 peripheral concentrations with the patients' survival. However, the small number of patients enrolled, the lack of adjustment with the disease severity and the inability of LTB4 plasma levels to be an independent predictive marker arise major concerns whether they could monitor disease behaviour and predict ARDS development in at risk patients (Table 6).

### Ferritin (Table 6)

Ferritin is a 480-kDa iron-storage protein that sequesters iron in the ferric (Fe^3+^) state. It has been speculated that ferritin may serve as a crucial antioxidant mediator because free iron enhances the formation of highly toxic hydroxyl radicals from superoxide anion and hydrogen peroxide. On the other hand, oxidative stress is a condition commonly seen in disorders at risk for ARDS development such as sepsis. Hence, ferritin-derived iron may aggravate oxidative damage in critically ill patients, contributing to the pathologic abnormalities encountered in ARDS. Furthermore, proinflammatory cytokines such as IL-8, IL-6 and TNFa which are increased and presumably participating in the pathogenetic derangements of ARDS have been suggested to promote ferritin synthesis [39,65]. Thereby, it can be concluded that elevated ferritin levels could result from oxidative stress, proinflammatory cytokines and the degree of lung injury, all conditions characterizing the pathogenesis of ARDS and subsequently can be used as prognostic and monitoring tool reflecting the likelihood of ARDS development and the disease severity.

The first study attempted to prove such correlation was conducted by Connelly et al [66]. They plotted ROC curves to estimate the utility of ferritin levels as prognostic factors and produced clinically useful cut-off points which could predict the development of ARDS with high sensitivity, specificity, negative and positive predictive value, both in male and female predominantly septic subjects. However, the heterogeneity of the etiologic factors resulting to ARDS development in at risk patients renders major uncertainty to the rigidity and reliability of the results.

To ameliorate this hardship, the same group of authors {Sharkey et al. [67]} generalized and extended the latter results in a homogeneous group of at-risk patients with multiple trauma demonstrating a strong correlation of initial ferritin plasma levels with the development of ARDS and multiple organ failure. In addition, an association of serum ferritin levels with the severity of lung injury as well as other markers of endothelial activation was also noted supporting the premise that elevated levels could reflect the inflammatory status encountering in ALI. Nonetheless, authors failed to detect specific predictive cut-off values suggesting that circulating concentrations of this biomarker are unable to predict per se the progression to ARDS. A possible explanation could arise from the hypothesis that elevated levels of this marker must reflect a systemic response to a risk factor, which may prove to reduce its specificity (Table 6).

## Lung epithelium-specific proteins (Table 7)

**Table 7:** Studies measuring lung-specific proteins in patients with or at risk for ARDS

**Investigator**	**Patients Controls**	**Biomarker / Summary**	**ROC curve analysis Cut-off values**	**Specificity – Sensitivity Diagnostic accuracy**	**Limitations**
Doyle et al. ^81^	15 ARDS^a^ 10 at risk 10 controls	SP-A is an acute indicator of lung function and alveolocapillary membrane injury	No	Not estimated	Small number of patients No ROC curve analysis / cut-off values No definitive relation with disease severity
Doyle et al. ^82^	22 ARDS^a^ 10 at risk 33 controls	Superiority of SP-B compared to SP-A plasma levels as a marker of lung function and alveolocapillary membrane injury	No	Not estimated	Only 3 case-control studies Inadequate sample size Lack of adjustment with disease behaviour No ROC curve analysis / cut-off values
Greene et al. ^83^	41 ARDS^a^ 22 at risk 35 controls	SP-A, SP-B, SP-D Serum changes found to be neither sensitive nor specific in predicting the onset of ARDS and discriminating survivors from non-survivors.	Yes Not reported	Poor predictive value Low specificity/sensitivity	Limited number of patients Serial measurements for a short period of time/ Lack of serial measurement for the most severe forms Heterogeneity of studied population Poor predictive value for serum levels
Cheng et al. ^84^	36 ARDS^a^ 2 ALI	SP-A levels were associated with severity of clinical lung injury and with disease outcome	No	Not estimated	Small sample size Causal diversity of studied population No serial measurement
Greene et al. ^85^	51 at risk 26 ARDS^a^ 16 controls	SP-A levels are predictive for at risk patients who developed ARDS from sepsis and aspiration but not trauma	No	Not estimated	Small sample size No ROC curve analysis / cut-off levels
Bersten et al. ^86^	54 at risk 9 controls	SP-B but not SP-A cut-off plasma levels predict ARDS development, particularly in at-risk patients suffering a direct lung injury	Yes SP-B: 4.994 ng/ml	78-85-85-78%	Small number of patients Limited follow-up serum data Most of patients had already lung injury Exclusion of milder at risk patients
Eisner et al. ^87^	565 ALI/ ARDS^a^	SP-A, SP-D Attenuation of SP-D plasma levels by lower volume ventilation strategies	No	Not estimated	Only 2 serial measurements Heterogeneity of studied population Potential selection bias No ROC curve analysis / cut-off levels
Ishizaka et al. ^95^	35 at risk 27 ARDS^a^ 21 controls	Association of optimal cut-off values of KL-6 serum levels with patients' mortality	Yes KL-6: 253 U/ml	100-87%	Inadequate sample volume Heterogeneity of studied population
Sato et al. ^96^	28 ARDS^a^ 10 controls	Association of KL-6 serum levels with variables of lung injury severity and with mortality rates No correlation with ventilation strategies	No	Not estimated	Small sample size Heterogeneity of studied group No serial measurement No ROC curve analysis / cut-off levels Diversity of ventilatory treatment

Abbreviations: ALI: Acute Lung Injury, ARDS: Acute Respiratory Distress Syndrome, BAL: Bronchoalveolar Lavage, KL-6: Krebs von den Lungen-6, ROC: Receiver Operating Characteristic, SP: Surfactant Protein, ^a: ^Use the American European Consensus Conference definitions

Beyond other important functions, the lung epithelium produces complex secretions, including mucus blanket, surfactant proteins, as well as several proteins important for host defense [68].

Sampling the epithelial lining fluid by bronchoalveolar lavage (BAL) represents the common means of studying the proteins secreted by the lung epithelium and investigating their alterations in lung disorders [69]. However, the past fifteen years pioneering studies [70] showed the presence of these proteins in the bloodstream as well, even though in small amounts. Because these proteins are mainly, if not exclusively secreted within the respiratory tract, their occurrence in the vascular compartment can be explained by several hypothetical mechanisms including, leakage from the lung into the bloodstream, increased production by the alveolar type II cells or diminished clearance rates from the circulation [68].

### Surfactant-associated Proteins

Pulmonary surfactant is a complex and highly surface active material covering the alveolar space of the lung. Biochemically, surfactant is a molecular mixture composed mainly of structurally heterogeneous phospholipids. A major function of pulmonary surfactant is to reduce the surface tension at the air-liquid interface of the alveolus, thereby preventing alveolar collapse on expiration. It has also been demonstrated that the surfactant contains specific proteins [71]. Four surfactant-specific proteins with different structural and functional properties have so far been identified. They were named surfactant protein-(SP)-A, SP-B, SP-C and SP-D according to the chronologic order of their discovery [72] and have been divided in two distinctive groups, the low-molecular-weight hydrophobic SP-B and SP-C and the high-molecular-weight-hydrophilic SP-A and SP-D. The latter belong to the collectin subgroup of the C-type lectin superfamily and are produced by two types of non-ciliated epithelial cells in the peripheral airway, Clara cells and alveolar type II cells. Studies have demonstrated that SP-B and SP-C seem to play an essential role for the adsorption of phospholipids to the air-water interface resulting to a stable phospholipids film and for the dynamic surface-tension-lowering properties [73]. Additional functions of the alveolar surfactant system include prevention of alveolar edema [74] and a pronounced influence, especially of the collectins SP-A and SP-D in the innate immune system of the lung [75,76] and have been used as useful markers for confirming the diagnosis and evaluation of disease activity of various ILDs since they reflect the epithelial damage and turnover [77]. Thus, it has been speculated that alterations of SPs in biological fluids could serve as valuable markers of the severity of the lung injury or clinical outcome in ARDS patients.

Most of our knowledge regarding changes in SP concentrations that occur in patients with or at risk for ARDS and their value in reflecting the disease severity or the likelihood of ARDS development comes from BAL studies. Several reports in the literature have demonstrated the occurrence of low SP-A levels in BALF of patients with ARDS following trauma [78,79] coupled with a strong relation of this biological marker to the severity of endothelial damage [80]. Moreover, a potential value of SP-A plasma levels in discriminating patients with ALI of various etiologic factors has also been shown [79]. On the other hand, little is known about changes in peripheral concentrations of surfactant-associated proteins in patients with ALI and whether these alterations can serve as markers of injury to the epithelial and endothelial barriers in the lungs.

Doyle et al. [81] documented elevated circulating concentrations of SP-A in patients with ARDS and in those with acute cardiogenic pulmonary edema possibly resulting from increased alveolocapillary permeability due to excessively high pulmonary capillary pressures. In the same study, blood SP-A levels were inversely associated with blood oxygenation and static respiratory system compliance. These results were fully confirmed by the same group of authors {Doyle et al. [82]} who also illustrated a clear superiority of SP-B compared to SP-A plasma levels as a marker of lung function and alveolocapillary membrane injury.

Another study by Greene et al. [83], evaluated the differences that occur in SPs in BALF and serum of a relatively small cohort of patients at risk for ARDS and during the course of the syndrome. Authors demonstrated that only SP-A and SP-D BALF levels were strongly related to outcome and likelihood of disease progression whereas serum changes found to be neither sensitive nor specific in predicting the onset of ARDS and discriminating survivors from non-survivors.

These data were further confirmed by a small cohort observational study by Cheng et al [84]. Even though authors reported an association of elevated SP-A plasma levels with a high degree of lung injury, they failed to extend this correlation with the disease mortality. Moreover, serum SP-D levels exhibited weak relation to the disease severity. It should also be noted that the aforementioned results present low statistical power due to the limited number of patients, the causal heterogeneity of the studied group and the absence of serial measurement and therefore no meaningful outcome can be excluded.

In harmony with the latter results, Greene et al. [85] found that plasma SP-A was weakly predictive for ARDS development in septic patients and were unable to detect at risk trauma patients that developed the syndrome. Additionally, authors raised the crucial issue whether circulating SPs can reflect pathophysiologic differences between direct and indirect causes of ARDS and subsequently detect biologic changes early after an insult. From a pragmatic clinical perspective the most important question to be answered is which ICU patients requiring ventilatory assistance will develop ARDS.

To do so Bersten et al. [86] generated ROC curve analysis and identified practical thresholds for SP-B plasma levels that could be clinically useful in predicting ARDS development, particularly in at-risk patients suffering a direct lung injury. In consistency with earlier studies [83-85] SP-A blood levels added no significant information on the disease prognosis. Further, an increase of circulating SP-B concentrations was documented on study entry, before changes in commonly applied clinical variables for the assessment of lung injury become apparent. These findings emphasize the usefulness of surfactant-associated proteins for the early detection of ARDS pathophysiologic alterations preceding changes in clinical parameters such as respiratory dysfunction. Arguments that can be made include the small sample size, the limited sequential measurements and the exclusion from the study recruitment of at risk patients with milder pulmonary dysfunction. These caveats coupled with the evidence that a considerable number of patients studied had already lung injury, pose major limitations to the predictive capacity of SP-B plasma levels and raise the necessity for larger prospective studies.

The only so far large multicenter randomized controlled trial was performed by Eisner et al. [87] who estimated the prognostic value of SP-A and SP-D levels in an overall of 565 patients with early ALI/ARDS. Authors conducted the first study with adequate statistical power to examine the impact of SPs on mortality and other clinical variables and clearly demonstrated a strong linkage of elevated SP-D levels with worse clinical outcomes such as greater risk of death, fewer ventilator- free and organ failure-free days. One of the most remarkable ascertainments of this study was the attenuation of SP-D plasma levels by the lower volume ventilation strategies which reduces patients' mortality postulating for the first time a significant association of biological parameters with therapeutic approaches and subsequently emphasizing the role of this mediator as a marker of the disease severity and prognosis. However, this study exhibited substantial weaknesses including the lack of sufficient serial measurements, a potential selection bias of patients recruited and the diversity of predisposing factors for ARDS development. These observations are not to diminish their value as prognostic and monitoring tools but to highlight the need for further confirmation studies using independent and well-defined populations of ALI/ARDS patients.

### Mucin-associated Antigens

Mucins are major components of the mucus layer covering the airway epithelium. They consist of high-molecular-weight glycoproteins belonging to a broad family of mucin peptides [68]. Mucins are either associated with membranes or secreted at the surface of the respiratory tract [68]. Krebs von den Lungen-(KL)-6 is mainly associated with cellular membranes. It was initially described by Kohno et al. [70] as a high-molecular-weight glycoprotein and was classified as human MUC1 mucin. Immunohistochemistry has mainly detected KL-6 in alveolar type II and epithelial cells of the respiratory bronchioles. KL-6 is predominantly expressed by airway cells; however, is not entirely lung specific, since it is also present on other somatic cells, such as pancreatic cells, eosophageal cells and fundic cells of the stomach [88]. Additionally, KL-6 is a sensitive indicator of damage to alveolar type II cells, which strongly express this mucin at their surface [70]. Type II pneumonocytes are regenerated over the alveolar basement membrane after the death of type I pneumonocytes over the first stage of lung injury. Therefore, its raise would theoretically represent the destruction of the normal lung parenchyma and architecture, the increased permeability of the air-blood barrier as long as the regenerating process as expressed by type II pneumonocytes' activity.

Towards this direction, the presence of KL-6 has been extensively used with great promises to monitor the severity of disease in idiopathic pulmonary fibrosis [89-91] and other interstitial lung diseases [92-94]. Since damage to, and disruption of, the alveolar epithelial lining coupled with loss of integrity of the air-blood barrier represent key features in the pathophysiology of ARDS, KL-6 serum levels could potentially serve as valuable indicators of the disease severity directly assessing the degree of epithelial damage and predicting the progression to ARDS. Nevertheless, only few studies so far, have evaluated their monitoring and prognostic efficacy in patients with or at risk for ARDS development.

One of the first studies to do so was recently carried out by Ishizaka and co-workers [95]. Authors generated ROC curve analysis and documented a highly sensitive and specific association of optimal cut-off values of KL-6 serum levels with patients' mortality. The latter, further supports the premise that disruption of the alveolar barrier represents a major determinant of prognosis of ALI and that serial measurements of KL-6 plasma levels might be helpful markers of the disease progression. Limitations that should be addressed include the limited number of patients, the retrospective analysis of the results and the diversity of the etiologic factors of ALI generate major concerns about the reproducibility and the reliability of the data.

Recently, Sato et al. [96] sought to determine potential correlations of KL-6 circulating concentrations with disease severity, patients' survival and different predisposing factors of ARDS. The most remarkable ascertainments of this study include strong associations of KL-6 peripheral levels with variables of lung injury severity and with the rates of mortality indicating possible relationship between the degree of epithelial damage and poor outcome in ARDS. Even though, authors attempted to show a modulation of KL-6 serum levels by ventilatory strategies, this relationship failed to reach a statistical significance. Despite substantial weaknesses exhibited such as the lack of serial measurement, the small sample size and the diversity of the applied treatment data derived from this study is highly informative and provides important knowledge regarding the biological impact of mechanical support strategies in this syndrome indicating the monitoring value of the epithelial damage markers. Further and sizeable prospective studies are required to validate the aforementioned hypothesis (Table 7).

## Future challenges and limitations

The ARDS represents an overwhelming inflammatory reaction to numerous insults within the pulmonary parenchyma resulting in life-threatening derangements in pulmonary vasomotion, alveolar ventilation and gas exchange. ARDS is a frequent disease with a devastating incidence between 13.5 and 75 per 100,000, thus affecting about 16–18% of all patients ventilated in the ICU [76,97]. Hence, ALI/ARDS is a major public health problem encountered frequently by all physicians who care for critically ill patients. Despite the fact that research efforts over the past several years have provided a more comprehensive knowledge of the potential mechanisms comprising the immunopathogenesis of ALI/ARDS and led to the development of innumerable causative or symptomatic treatment approaches, the mortality rate of these patients remains unacceptably high at 30–40% [97]. Currently, the only therapy that has been proven to be effective at reducing mortality is a protective ventilatory strategy [98]. However, new therapies are still needed. One of the most fruitful applications is monitoring the disease activity and consequently the early identification of at risk patients with increased likelihood of non-response to treatment and progression to ARDS. Nevertheless, there are problems with the sensitivity, effort-dependability and ease of repetition of the current modalities being used for this purpose, including radiological and BAL techniques as well as clinical and physiological indices of pulmonary injury (Murray score), systemic illness (Acute Physiology and Chronic Health Evaluation- APACHE-II score and Simplified Acute Physiological Score -SAPS), ARDS severity (respiratory system compliance and PaO2/FiO2 ratio) and multiorgan system failure (Multi-Organ Dysfunction Score-MODS). Most of these clinical parameters have failed to be independent predictors of mortality in studies of adults with ALI/ARDS [99,100]. Development of a prognostic index that combines clinical and biological determinants may be useful to ameliorate these hardships.

On the basis of this conception, a large body of serum markers either cytokines and lung-specific proteins or markers of endothelium and neutrophil activation as well as other serological parameters probing different facets of the immunopathogenesis of ALI/ARDS has been delineated. The applications of these markers in the clinical setting created major expectations in terms of defining categories of patients for different therapies or prognosis for the purpose of counseling families and patients and/or possibly identifying novel therapeutic targets. The determination of a reliable serologic marker reflecting the disease behaviour and adding independent information regarding the development of the syndrome before it becomes obvious in clinical level, easily reproducible and feasible to be measured serially represents a major challenge. The early serial measurement of this biomarker may serve as an independent non-invasive prognosticator of the disease outcome even at the onset of the syndrome and therefore lead to an early detection of at risk patients with increased likelihood of progression to ARDS. The latter, if sufficiently accurate, could prove extremely useful in identifying and counseling families of patients at low or high risk for adverse outcomes and further, will allow ventilatory or other types of treatment to be evaluated or eventually modulated before they have failed in the high risk group. The presented data give credence to the view that multiple biomarkers can be used to measure the lung and systemic response to a protective ventilatory strategy and potentially to discriminate patients who are ineffectively treated and might be candidates for rescue therapies [49,87].

More importantly, use of one or more of these biologic markers to select a group of patients at higher risk of adverse clinical outcome could be used to restrict or stratify enrollment in future clinical trials applying novel ventilator treatments such as high-frequency oscillatory ventilation leading to a better patient care. Thereby, a combination of clinical factors and biologic marker measurements could be crucial for the selection of more homogeneous groups of patients with ALI/ARDS for further studies producing evidence of high scientific rigidity [101]. Finally, understanding the relative roles of markers of systemic and pulmonary endothelial injury and other inflammatory mediators to the pathogenetic process of the syndrome is likely to lead to valuable insights into the final pathway resulting in diffuse alveolar damage and significant lung dysfunction, highlighting therapeutic targets for novel interventions. The aforementioned components can potentially compile a clinician's "wish list".

However, the feeling of excitement arising from the expected clinical utility comes in contrast with important deficiencies exhibited by the new methodologies including non-standardization techniques, lack of knowledge of reproducibility and link to disease behaviour. Furthermore, most of the studies enrolled a limited number of patients, insufficient to extract any meaningful or statistically significant outcome. In addition, the heterogeneity of the studied population resulting from the causal diversity of the syndrome and the use of non-uniform criteria for the definitions of ARDS and at-risk patients (Tables 2, 3, 4, 5) render major uncertainty to the reproducibility and the scientific rigidity of these findings and may explain potential discrepancies between various studies investigating different groups of at risk patients.

Moreover, many of the caveats arising from this data are generated by the origin disadvantages of the investigated serological parameters to serve as specific markers of the disease activity and severity. In particular, it is well known that assays of circulating cytokine concentration may be misleading, because they do not detect receptor or cellular bound cytokines, or they may fail to detect cytokines when inhibitors or receptor antagonists are present. Thus, measured cytokine concentrations may not reflect the disease activity or the state of inflammation but the increased production or decreased clearance from the circulation. In consistency with these limitations, it should be underlined that cytokines are part of an inflammatory cascade and biological effects are difficult even impossible to interpret without the appreciation of the entire network of the inflammatory response. Hence, data in most of the studies was inconclusive and incomplete since none of them analyzed serum alterations of a considerable number of inflammatory components.

Additionally, it is of high importance to note that unfortunately only few studied molecules (VWF, IL-1β, IL-6, ICAM-1, VCAM-1) exhibited independent discriminatory power [18,44,48,49] and associated with the mortality of patients with high sensitivity and specificity [45]. Finally, only the minority of the studies [18,19,25-27,43,45,47,51,53,64,66,67,83,86,95] clarified the effectiveness and the diagnostic accuracy of the biomarkers by applying ROC curve analysis which is essential to estimate the sensitivity and specificity of a marker and to introduce clinically practical cut-off levels for the prediction of ARDS development in at risk individuals.

Collectively, these findings highlight the necessity for further investigations in the context of large prospective studies analyzing homogeneous and well defined group of ARDS or at risk patients and the assessment of novel molecules to serve as diagnostic and prognostic tools, as well as markers of the disease activity and severity.

## Conclusion

Currently, the application status in routine clinical practice for most of these biologic markers is still in its infancy and remains exploratory. Unfortunately, they do not yield independent indications for therapy or mark the end of the inflammatory process and their prognostic value still needs to be established. Although the majority of them have not yet lived up to the "great hype" that was generated, markers of endothelium activation and mostly VWF and adhesion molecules (ICAM-1, VCAM-1, E-selectin) show the greatest promise in ARDS and ALI. On the contrary the majority of serum cytokines and ferritin appear to be not ready for routine monitoring since they may reflect an inflammatory response to a risk factor rather than lung injury and disease severity. Additionally, lung specific proteins have proven to be neither specific nor sensitive for the prediction of ARDS development and the disease outcome and moreover they have failed to associate with alterations in the ventilatory strategies in large clinical trials. Further prospective investigations, technical improvements and introduction of novel markers are warranted in order to elevate the association of serum biomarkers with the pathogenesis of ARDS in the same status as for tumour markers with lung cancer. Nevertheless, crossing the boundary from research to clinical application requires validation in multiple settings, experimental evidence supporting a pathophysiologic role, and ideally intervention trials showing that modification improves the outcome. The emergence of pioneering technologies including DNA microarrays which have already been applied with great success in the respiratory research field [102] can help scientists to circumvent this problem and bridge this boundary. In the interim, these markers can be quite useful to supplement the clinical, radiological and physiological monitoring of the disease and identify high-risk patients who would benefit from aggressive management of established risk factors.

## List of Abbreviations

Acute Lung Injury (ALI)

Acute Physiology and Chronic Health Evaluation (APACHE)

Acute Respiratory Distress Syndrome (ARDS)

Bronchoalveolar lavage fluid (BALF)

ICU: Intensive Care Unit

ICAM-1: Intercellular Cell Adhesion Molecule-1

ILs: Interleukins

ILDs: Interstitial Lung Diseases

Krebs von den Lungen-(KL)-6

LTs: Leukotrienes

MMPs (Metalloproteinases)

MODS: Multi-Organ Dysfunction Score

Receiver-operating-characteristic (ROC)

Simplified Acute Physiological Score (SAPS)

SIRS: Systemic Inflammatory Response Syndrome

Soluble-E-selectin: s-E-selectin

Soluble IL-2 receptor (sIL-2R)

Surfactant protein-(SP)

TNFa: Tumor Necrosis Factor-alpha

VWF: von Willebrand factor

VCAM-1: Vascular Cell Adhesion Molecule-1

## Competing interests

The author(s) declare that they have no competing interests.

## Authors' contributions

AT, IP and DB were involved with the study conception. AT and IP performed the data acquisition and interpretation. DB was involved in revising the article for important intellectual content. All authors read and approved the final manuscript.
